# Age-Related Acute Subjective, Behavioral, Cognitive and Cardiovascular Effects of Tetrahydrocannabinol

**DOI:** 10.1093/geroni/igaf122.1970

**Published:** 2025-12-31

**Authors:** Deepak Cyril D’Souza

**Affiliations:** Yale University, New Haven, Connecticut, United States

## Abstract

There has been a relatively recent increase in recreational and medical cannabis use in older adults compared to younger adults which is projected to further increase4 and is associated with legalization of cannabis. Further, there are higher odds of cannabis-related problems in those ≥55 years including a significant increase in emergency department visits for acute consequences of cannabis. However, although there is a wealth of studies of cannabis effects in younger populations, the acute effects of cannabinoids in older adults have not been well-studied. Little is known about their effects in older individuals despite growing use of cannabis in this demographic. Age-related changes in 1) the endocannabinoid system, the target of THC, 2) cognitive function, 3) psychomotor function, 4) postural stability, 5) cardiovascular reserve, and 6) drug metabolism may render older adults more vulnerable to THC and cannabis. Data from a controlled double-blind, placebo-controlled crossover study on the subjective, behavioral, cognitive, psychomotor and cardiovascular effects of THC in older vs. younger adults will be presented. Age-related differences in the pharmacokinetics of THC will also be explored. On immediate recall, older individuals recalled fewer words vs. younger controls under the influence of THC relative to placebo both on immediate and delayed recall. Furthermore, relative to younger individuals, older individuals reported greater THC-induced anxiogenic effects. These preliminary data support further research on the benefits and risks of cannabis an its principal active constituent THC, in the aging population.

